# The Involvement of Wheat F-Box Protein Gene *TaFBA1* in the Oxidative Stress Tolerance of Plants

**DOI:** 10.1371/journal.pone.0122117

**Published:** 2015-04-23

**Authors:** Shu-Mei Zhou, Xiang-Zhu Kong, Han-Han Kang, Xiu-Dong Sun, Wei Wang

**Affiliations:** 1 State Key Laboratory of Crop Biology, College of Life Science, Shandong Agricultural University, Tai’an, Shandong, People's Republic of China; 2 College of Horticulture Science and Engineering, Shandong Agricultural University, Tai’an, Shandong, People's Republic of China; University of Delhi South Campus, INDIA

## Abstract

As one of the largest gene families, F-box domain proteins have been found to play important roles in abiotic stress responses via the ubiquitin pathway. *TaFBA1* encodes a homologous F-box protein contained in E3 ubiquitin ligases. In our previous study, we found that the overexpression of *TaFBA1* enhanced drought tolerance in transgenic plants. To investigate the mechanisms involved, in this study, we investigated the tolerance of the transgenic plants to oxidative stress. Methyl viologen was used to induce oxidative stress conditions. Real-time PCR and western blot analysis revealed that TaFBA1 expression was up-regulated by oxidative stress treatments. Under oxidative stress conditions, the transgenic tobacco plants showed a higher germination rate, higher root length and less growth inhibition than wild type (WT). The enhanced oxidative stress tolerance of the transgenic plants was also indicated by lower reactive oxygen species (ROS) accumulation, malondialdehyde (MDA) content and cell membrane damage under oxidative stress compared with WT. Higher activities of antioxidant enzymes, including superoxide dismutase (SOD), catalase (CAT), ascorbate peroxidase (APX) and peroxidase (POD), were observed in the transgenic plants than those in WT, which may be related to the upregulated expression of some antioxidant genes via the overexpression of TaFBA1. In others, some stress responsive elements were found in the promoter region of *TaFBA1*, and TaFBA1 was located in the nucleus, cytoplasm and plasma membrane. These results suggest that *TaFBA1* plays an important role in the oxidative stress tolerance of plants. This is important for understanding the functions of F-box proteins in plants’ tolerance to multiple stress conditions.

## Introduction

Reactive oxygen species (ROS), including the superoxide anion radical (O_2_^−^), the hydroxyl radical (OH^•^) and hydrogen peroxide (H_2_O_2_), are products of normal metabolic reactions in cells and are usually formed at low levels. However, under conditions of various environmental stresses, such as salinity, drought and extreme temperature, the ROS levels tend to increase in plant cells [1, 2]. The overproduction of ROS in plants causes oxidative damage to DNA, pigments, proteins and lipids, and it subsequently leads to a series of destructive processes [3, 4]. Therefore, oxidative stress is the most universal second stress involved in almost all stress conditions [5], and it is also the common mechanism by which abiotic stresses affect plant growth and development. To protect themselves against ROS, plants have developed a combination of enzymatic and non-enzymatic antioxidative mechanisms [3, 6–7].

The ubiquitin 26S proteasome system (UPS) is important for the quality control of intracellular proteins and has emerged as a major player in plant responses to abiotic stresses [8]. In the UPS, the protein modified by an ubiquitin chain is subsequently degraded by the 26S proteasome. Three enzymes are involved in the ubiquitination of a target protein, including E1 ubiquitin-activating enzyme, E2 ubiquitin-conjugating enzyme and E3 ubiquitin ligase. Among these, E3 is the key enzyme that defines the specificity of the target proteins [9]. The E3 ligase group is a far more diverse group and can be divided into different families based on known E3 ligase motifs: homologous to E6-AP C terminus (HECT), Ring/U-box and anaphase-promoting complex (APC) and Skp1-Cullin-F-box complex (SCF) [10].

As a major subunit of the SCF complex, the F-box protein, which is characterized by a conserved 40-50-amino acid F-box motif, works as a determinant in substrate recognition and interacts with Skp1 through the F-box motif at the N-terminus of the protein [11]. Several F-box proteins have been characterized that play important roles in responses to (a)biotic stresses [12, 13].

Previously, we isolated the F-box gene *TaFBA1* from wheat (*Triticum aestivum* L.) [14]. We found that the drought tolerance of the transgenic plants with overexpressed *TaFBA1* was improved. To understand the underlying mechanisms, we investigated the involvement of antioxidative competition of the transgenic plants in this study. The results indicated that the levels of reactive oxygen species (ROS) accumulation, MDA content, and cell membrane damage were less in the transgenic plants than in WT under oxidative stress, suggesting improved antioxidative capability in the transgenic plants. Enhanced antioxidant enzyme activities and gene expression may be involved. These results are important to understand the functions of *TaFBA1* in plant stress tolerance.

## Materials and Methods

### Plant material and treatments

Wheat (*Triticum aestivum* L. cv shannong 16) seedlings were cultivated according to Zhou et al. [14] with some modifications. The oxidative stress treatments were induced by methyl viologen (MV) with sterile water as a control. Wheat seedlings with one leaf were subjected to different oxidative stresses and harvested at different time points after treatment.

The transgenic tobacco plants were produced and identified as described previously by Zhou et al. [14]. Three transgenic tobacco lines, OE-3, OE-5 and OE-6, were used. To detect the seed germination after MV treatment, tobacco seeds from transgenic and WT plants were surface-sterilized and sown according to Zhou et al. [14]. The number of germinated seeds was counted.

For MV treatment on young seedlings, the 7-d-old tobacco seedlings were grown on MS medium containing 0, 5 or 10 μM MV for 7 d. The corresponding fresh weights and root lengths were measured.

MV damage was then analyzed using the leaf disks experiment as described by Yun *et al*. [15], with slight modifications. Leaf disks (7-mm diameter) from the third leaves of WT and transgenic plants grown in a greenhouse for two months were transferred to 5.0-cm Petri dishes containing 5 ml of MV solution at various concentrations (0, 50, 100 μM). Fifteen leaf disks were placed in each Petri dish and were then incubated at 25°C for 48 h under continuous white light at 100 μmol s^−1^ m^−2^. The effect of MV on leaf disks was analyzed by observing the phenotypic changes and by measuring the chlorophyll content, malondialdehyde (MDA) content and relative electrical conductivity.

At the whole grown plant level, the 90-d-old plants of each line (six plants per line) were sprayed with MV solution (50, 100 μM) containing 0.1% Tween-20 and then transferred to an illuminated incubation chamber (GXZ-260C) with a photon flux density of approximately 100 μmol m^−2^ s^−1^ for 48 h.

### Gene expression analysis by Real-time RT-PCR

Total RNA was extracted from the wheat or tobacco leaves with Trizol reagent (TaKaRa, Dalian, China) according to the manufacturers’ protocol. The total RNA was subjected to first-strand cDNA synthesis with ReverAid First Strand cDNA Synthesis Kit (Fermentas, USA) according to the manufacturers’ protocol. *TaFBA1* expression was followed by a 222-bp fragment amplified with the specific primers QFBA1 and QFBA2. The α-tubulin cDNA was used as a control reference. Quantitative analysis was performed using the Bio Rad CFX Manager system with the following program: 95°C for 15 s, then 40 cycles of 95°C for 15 s, 58°C for 30 s and 72°C for 15 s. The absence of prime-dimer formation was confirmed using single and primerless controls. The relative transcript abundance for some genes derived from NCBI was relative to the actin transcript levels measured in the same sample. The primers used for qPCR are listed in Table 1.

**Table 1:** Primer sequences were used in the article. (S = C/G, W = A/T, N = A/C/G/T).

Names	Sequence(5’-3’)	Length(bp)
**Tubulin-1**	ATCTGTGCCTTGACCGTATCAGG	23
**Tubulin-2**	GACATCAACATTCAGGACACCATC	24
**FBA5**	TCTAGAGAGATGGAAGAGCACCGTGAGGA	29
**FBA6**	GGTACCTGGAGACTCCGGCCAGTCGA	26
**QFBA1**	AGCAGCAGAACAAGCCTGACCA	22
**QFBA2**	ACGTGACGTTGGACAGCCTTTG	22
**Ntactin-F(U60489)**	CATTGGCGCTGAGAGATTCC	20
**Ntactin-R(U60489)**	GCAGCTTCCATTCCGATCA	19
**NtSOD-F (AB093097)**	GACGGACCTTAGCAACAGG	19
**NtSOD-R(AB093097)**	CTGTAAGTAGTATGCATGTTC	21
**NtCA-F (AF454759)**	CGCCTGTGGAGGTATCAAA	19
**NtCA-R (AF454759)**	GAGAAGGAGAAAGACCGAACT	21
**NtRbohD-F(AJ309006)**	ACCAGCACTGACCAAAGAA	19
**NtRbohD-R(AJ309006)**	TAGCATCACAACCACAACTA	20
**NtCAT1-F (U93244)**	TGGATCTCATACTGGTCTCA	20
**NtCAT1-R (U93244)**	TTCCATTGTTTCAGTCATTCA	21
**NtGPX-F (AB041518)**	GGTTTGCACTCGCTTCAAG	19
**NtGPX-F (AB041518)**	AGTAGTGGCAAAACAGGAAG	20
**NtAPX1-F(AU15933)**	GAGAAATATGCTGCGGATGA	20
**NtAPX1-R(AU15933)**	CGTCTAATAACAGCTGCCAA	20
**NtAPX2-F (D85912)**	GACAACTCATACTTTACGGA	20
**NtAPX2-R (D85912)**	CTTCAGCAAATCCCAACTCA	20
**AP1**	NTCGASTWTSGWTT	14
**AP2**	NGTCGASWGANAWGAA	16
**AP3**	WGTGNAGWANCANAGA	16
**AP4**	TGWGNAGSANCASAGA	16
**AP5**	AGWGNAGWANCAWAGG	16
**AP6**	STTGNTASTNCTNTGC	16
**GSP1**	CATAGAAACGACCAGGACGACAGACACAG	29
**GSP2**	AACCCACAGAGAAGCCGCTCCATTGACAG	29
**GSP3**	ATGCCCTCCTTGTCCTGGATTTTCGCCTT	29
**GSP21**	CGGAAGCTCCAGGGTCTTGAGAGATCGC	28
**GSP22**	GGTGAGCTGGTCAGGCTTGTTCTGCTGC	28
**GSP23**	ATAGGTCCTCCCAGCATCTTTCCTCACG	28

### Detection of protein carbonylation by immunoblotting

Leaf tissue (0.5 g fresh weight per ml) was ground in a chilled mortar in protein extraction buffer containing 100 mM Tris-HCl (pH 8.0), 10 mM β-mercaptoethanol, 1 mM EDTA, 0.4 M sucrose, 1 mM PMSF, 5 mg l^-1^ leupeptin, 5 mg l^-1^ aprotinin, 5 mg l^-1^ antipain and 10% streptomycin sulfate [16, 17]. Proteins were separated with SDS-PAGE on a 12% gel and transferred onto a polyvinylidene fluoride (PVDF) membrane (Millipore, Billerica, USA), followed by successive incubation in 2,4-dinitrophenylhydrazine (DNPH, 0.1 mg ml^-1^ in 2 M HCl) or 2 M HCl without DNPH (control) for 5 min [16]. Next, the membrane was washed three times in 2 M HCl and seven times in 100% methanol for 5 min each. Protein carbonylation was routinely detected with the anti-DNP antibody (Sigma, Tucson, USA).

### TaFBA1 protein analysis by immunoblotting

Total protein was extracted from tobacco leaves [18]. Protein content was determined by the dye-binding assay according to Bradford [19]. Proteins were separated with SDS-PAGE on a 12% gradient gel and transferred to polyvinylidene fluoride (PVDF) membranes (Millipore, Billerica, MA). Proteins were detected with the TaFBA1 antibody, which was produced as described previously by Zhou et al. [14].

### Measurement of the chlorophyll content, malondialdehyde content and relative electrical conductivity

The chlorophyll content was quantified according to the procedure of Hemavathi et al. [20]. MDA content was determined as described by Zhang et al. [21]. Relative electrical conductivity was determined as described previously [22].

### Extraction and assay of antioxidant enzyme

Superoxide dismutase (SOD; EC1.15.1.1), peroxidase (POD; EC 1.11.1.7), catalase (CAT; EC 1.11.1.6) and ascorbate peroxidase (APX; EC 1.11.1.1) activities were determined as described previously [23]. Enzyme activity assays were carried out using a UV-visible spectrophotometer (UV-2550, Shimadzu, Japan) at 25°C. The protein concentration of each enzyme extract was determined according to the method described by Bradford [19].

### Determination of H_2_O_2_ content, production rate of O_2_^·–^ and staining with DAB, NBT

The production rate of O_2_·^–^ and H_2_O_2_ content were determined according to Zhang et al. [23]. H_2_O_2_ content was visualized with diaminobenzidine (DAB), and O_2_·^–^ accumulation was visualized with nitroblue tetrazolium (NBT) according to Tian et al. [24].

### Cloning of the promoter region of *TaFBA1*

Based on the sequence of the *TaFBA1* gene, some reverse gene special primers (GSP) were designed. The primer sequences are shown in Table 1. *TaFBA1* gene promoter isolation was accomplished using the thermal asymmetric interlaced PCR (TAIL-PCR) technique as described by Terauchi and Kahl [25]. After two successful rounds of TAIL-PCR, we obtained an 1104-bp region upstream of the initiation codon (GenBank ID: **JQ065654**).

### Subcellular localization of TaFBA1

The open reading frame (ORF) of *TaFBA1* without a termination codon was obtained by PCR amplification using the specific primers FBA5 and FBA6 (Table 1). The PCR products were cloned into a pMD18-T simple vector (TaKaRa, Dalian, China) and digested by *Xbal* I and *Kpn* I. The fragments were fused to the N-terminus of the GFP expression vector PBI121 under control of the CaMV 35S promoter (35S::TaFBA1::GFP). The 35S::GFP was used as a control. Onion epidermal cells with 35S::TaFBA1::GFP were generated by agrobacterium-mediated transformation and they were then observed using a spectral confocal microscope (Olympus, Tokyo, Japan) with an excitation wavelength of 488 nm.

### Statistical analysis

All experiments and determinations were repeated a minimum of three times. To determine the relative electrical conductivity and activity of the antioxidant enzymes, the experiments were repeated at least six times. Statistical analyses were performed using SPSS and Excel, and *P*<0.05 or *P*<0.01 were considered to be statistically significant.

## Results

### *TaFBA1* promoter analysis

In our previous reports [14], a 978-bp full-length cDNA of *TaFBA1* was amplified by RT-PCR from wheat leaves. *TaFBA1* encodes 325 amino acids with a calculated molecular weight of 36.6 kDa. To characterize the expression of *TaFBA1*, the sequence of the *TaFBA1* promoter (GenBank accession no: JQ065654) was isolated and analyzed using the database search programs PLACE (http://www.dna.affrc.go.jp/PLACE/) [26] and PlantCARE (http://bioinformatics.psb.ugent.be/webtools/plantcare/html/) [27]. Several consensus *cis*-acting elements related to the stress response were found in the promoter region, such as MYB binding sites, anoxic specific inducibility responsive elements, wound-responsive elements, heat stress responsive elements and endosperm expression responsive elements (Table 2). In addition, ethylene-responsive elements, MeJA-responsive elements and salicylic acid responsive elements were also found. These suggested that the expression of *TaFBA1* is stress-inducible and may be involved in the response to multiple abiotic stresses.

**Table 2:** Putative *cis*-elements in the *TaFBA1* promoter.

*Cis*-element	Position	Sequence(5’-3’)
**MYB binding site**	-552, -192	TGACCG
**Anoxic specific inducibility responsive element**	-972, -766	CCCC CG
**Wound-responsive element**	-87	AGGAAATTT
**Heat stress responsive element**	-186, -221	AAATTTTTT
**Endosperm expression responsive element**	-142, -231	TGAGTCA
**Ethylene-responsive element**	-212	ATTTCAAA
**MeJA-responsive element**	-1020	CGTCA
**Salicylic acid responsive element**	-959	AAAAAGA CGG

### The response pattern of *TaFBA1* expression to oxidative stress in wheat

**Fig 1:**
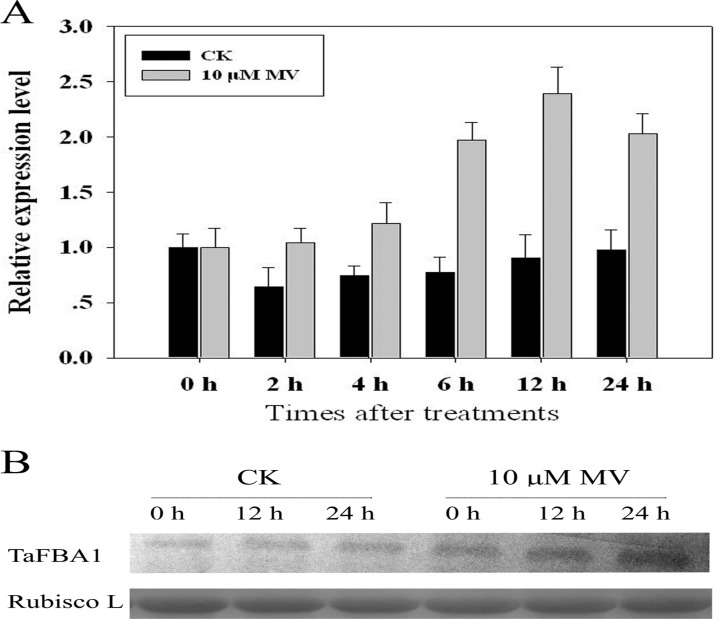
Expression of *TaFBA1* in wheat under MV-induced oxidative stress. Wheat seedlings with one leaf were subjected to 10-μM MV treatments. Seedlings treated with sterile water were chosen as controls. Seedlings were harvested at different time points for analysis. **(A)** Expression of *TaFBA1* at the mRNA transcript level in shoots, as shown by qPCR; tubulin cDNA was used as a control reference; **(B)** Expression of TaFBA1 at the protein level in shoots as shown by western blot. After 12.5% SDS-PAGE, protein samples were electro-transferred onto a PVDF membrane and probed with the TaFBA1 antibody produced in our laboratory. The Rubisco large subunit was used as a loading control.

To investigate the expression pattern of *TaFBA1* under oxidative stress, the wheat seedlings were treated with 10 μM MV. The mRNA transcript level was detected by real-time quantitative PCR. From Fig. 1A, *TaFBA1* mRNA was slightly up-regulated at 4 h after MV treatment, reached a maximum at 12 h and then returned. Further, the protein abundance of TaFBA1 was detected by western blot. As shown in Fig. 1B, TaFBA1 protein was obviously enhanced at 12 h and 24 h after MV treatment, but it showed no significant changes under normal conditions during this period. These results indicated that *TaFBA1* was oxidative stress responsive and may play roles in the plant’s oxidative stress tolerance.

### Effects of *TaFBA1* overexpression on the oxidative stress tolerance of transgenic plants

**Fig 2:**
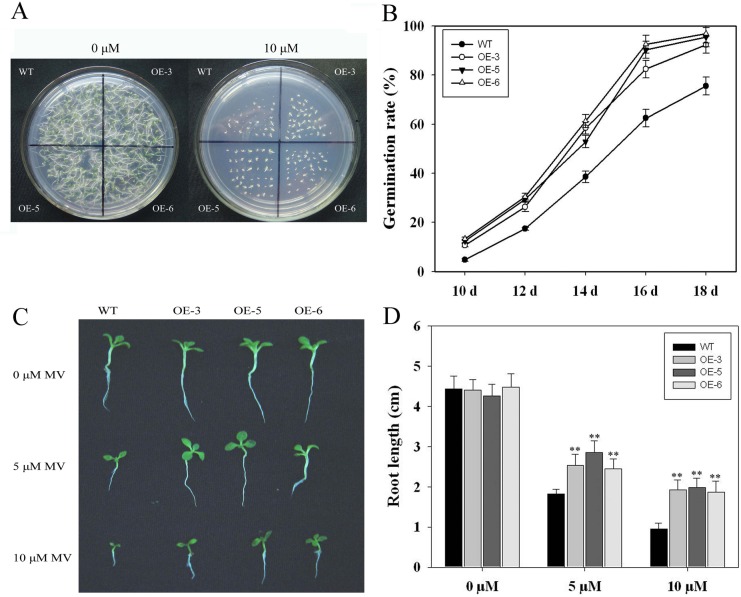
Germination and phenotype of different transgenic lines on MS medium containing different concentrations of MV. **(A)** Seed germination; **(B)** Germination rate. The seeds were allowed to grow for 16 d before the photographs were taken. Each measurement consisted of 50 seeds. Values are averages of three replicates ± *SE*. **(C)** Seedling development. **(D)** Root length. Seedlings grown on MS medium for 7 d were transferred to MS medium containing different concentrations of MV for 7 d before the photographs were taken. Root length was obtained from 30 seedlings in each of three independent experiments.

Seeds from WT and *TaFBA1*-transgenic lines (OE-3, OE-5 and OE-6) were germinated in culture media containing 10 μM MV. The results are shown in Fig. 2A and B. Under control conditions without MV, similar germination was observed between WT and *TaFBA1* transgenic plants. In the presence of 10 μM MV, *TaFBA1* seeds showed higher germination rates compared with WT seeds. The statistics data shown in Fig. 2B also indicated the faster germination of the transgenic tobacco seeds. After sowing for 10 d, the germination percentage of the transgenic seeds varied from 10.72–13.29%, while that of the WT was approximately 4.69%. When seeds were germinated for 18 d, the transgenic seeds had reached close to 96% germination, whereas the WT seeds were below 75% (S1 File). These results suggest that oxidative stress inhibited the seed germination, but less inhibition was observed in the transgenic plants than in the WT.

**Fig 3:**
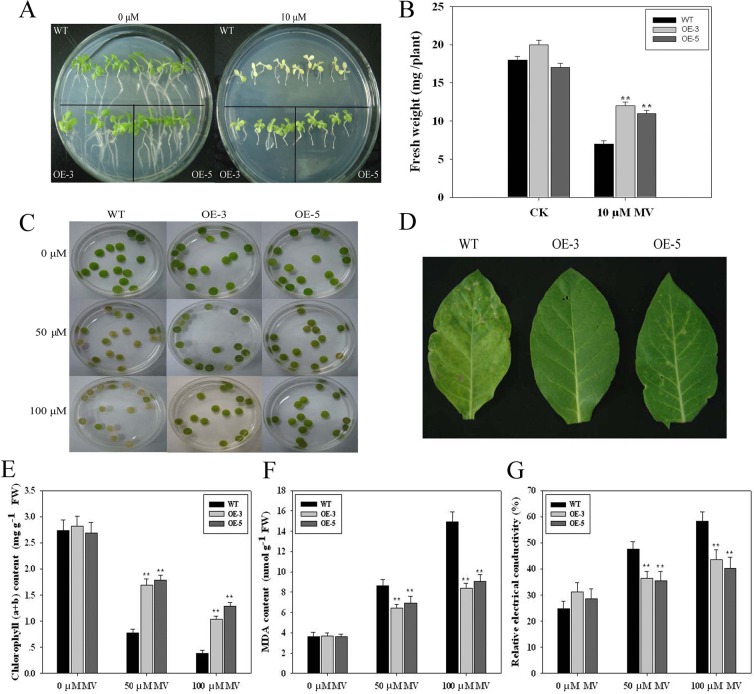
Methyl viologen-induced oxidative damage of leaves from WT and transgenic lines. **(A)** Phenotypes of WT and transgenic (OE-3, 5) seedlings in response to methyl viologen treatments. The corresponding fresh weights are shown in **(B).** Germinated seedlings with radicle lengths were transferred onto MS medium containing 0 or 10 μM methyl viologen for 7 d. **(C)** The phenotypic differences in the leaf disks from transgenic vs. WT plants after 24 h of MV treatment. **(D)** Leaf phenotype of three-month-old WT and transgenic tobacco plants treated with 100 μM MV for 48 h. Chlorophyll content (mg g-1 FW) **(E)**, malondialdehyde (MDA) content (mg g-1 FW) **(F)** and relative electrical conductivity (%) **(G)** in leaf disks of transgenic lines vs. WT plants floated on 0, 50 and 100 μM MV solution, respectively, for 48 h under continuous light at 25°C.

The growth responses of the transgenic seedlings to oxidative stress were also investigated, and the results are shown in Fig. 2C-D. In normal conditions without MV, no significant difference in plant size and root length was observed between WT and transgenic lines. However, when MV (5 or 10 μM) was applied to one-week-old seedlings for 7 d, the main root length in both types of seedlings was decreased, but less inhibition was observed in the transgenic plants than in WT (Fig. 2C and D). The data in Fig. 3A and Fig. 3B also indicated similar results as those in Fig. 2. After treatment with 10 μM MV for 7 d, serious chlorosis was observed in WT tobacco leaves, whereas the transgenic tobacco seedlings were still alive and green (Fig. 3A). Measurement of the fresh weight revealed that the transgenic plants had greater fresh weight than WT (Fig. 3B).

For the adult plants, we further tested the tolerance of leaf disks from two-month-old plants to exogenous MV. In the leaf disks assay, the phenotypic differences were observed from the leaf disks derived from WT and transgenic lines after 48 h MV (50 or 100 μ*M*) treatments. Severe necrosis was observed in the leaf disks of transgenic lines and WT plants treated with MV, but the situation was more serious in WT plants, whereas only partial necrosis was observed in transgenic lines (Fig. 3C). For elder two-month-old plants, a similar result was found after they were sprayed with 100 μM MV for 48 h. In comparison with WT, minor injured symptoms were observed in transgenic plants (Fig. 3D). The chlorophyll content of transgenic tobacco plants overexpressing TaFBA1 was significantly higher than that of the wild type under oxidative stress (Fig. 3E).

### Effects of oxidative stress on MDA content and relative electrical conductivity

We quantified the MDA content and relative electrical conductivity as a measure of cellular damage in response to oxidative stress. The transgenic plants showed a remarkably lower MDA content compared with WT after MV treatment, while it showed no significant difference under normal conditions (Fig. 3F). Relative electrical conductivity measurements displayed a pattern similar to MDA content and were lower in the transgenic lines relative to the WT under oxidative stress (Fig. 3G). The results suggest that transgenic plants experienced less lipid peroxidation and had a greater capability to maintain membrane stability under oxidative stress.

### Effects of oxidative stress on protein carbonylation

**Fig 4:**
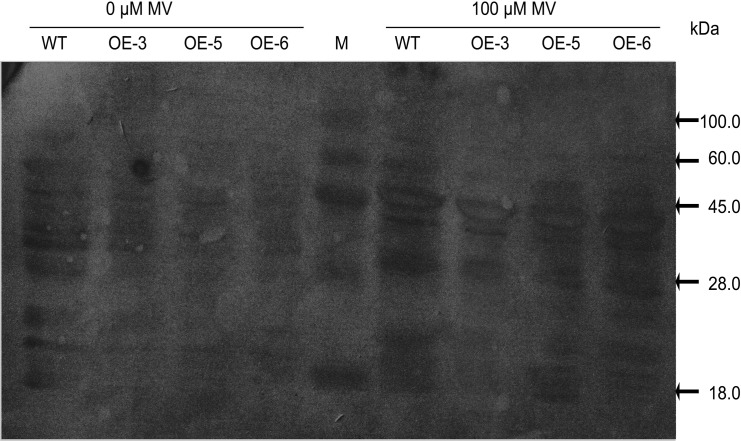
Protein carbonylation levels in WT and transgenic lines under oxidative stress. Protein carbonylation levels were detected with anti-DNP antibodies in WT and transgenic plant leaves under normal conditions or 100-μM MV treatments. Protein gel blot analysis of protein carbonylation following derivatization of protein carbonyls with DNPH in HCl.

Protein carbonylation was detected by immunoblotting (Fig. 4). Oxidative stress increased protein carbonylation in the leaves of both transgenic and WT plants. However, the amount of protein carbonylation products that accumulated in the WT was greater than that in transgenic plants under both normal and oxidative stress conditions. Meanwhile, in the control experiment using PVDF membranes treated with HCl only without 2, 4-dinitrophenylhydrazine (DNPH), no protein carbonylation was found (data not shown). This result suggests that less protein damage occurred in the transgenic tobacco plants than in WT under oxidative stress conditions.

### Effects of oxidative stress on ROS accumulation and antioxidative enzyme activities

**Fig 5:**
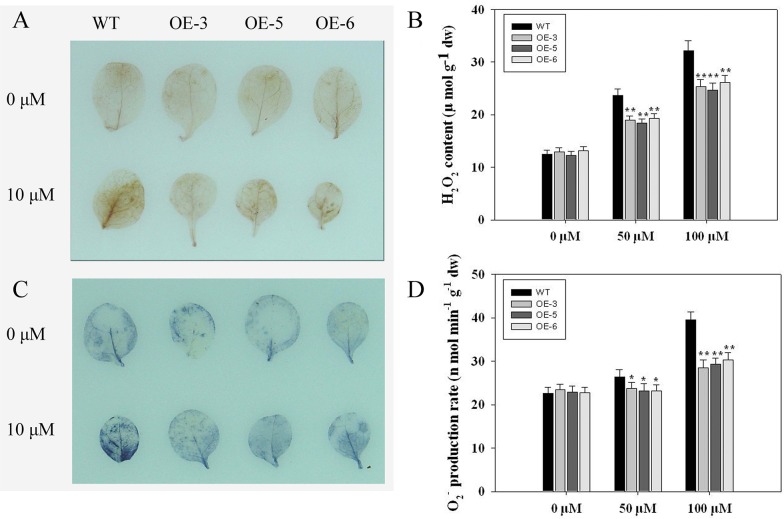
ROS accumulation in different transgenic plants and WT under MV-induced oxidative stress. **(A)** H_2_O_2_ accumulation detected by DAB staining; **(B)** H_2_O_2_ content_;_ **(C)** O_2_^.-^ accumulation detected by NBT staining; **(D)** O_2_^.-^ production rate_._ Values are averages of three replicates ± *SE*.

To examine whether the enhanced tolerance of oxidative stress is associated with ROS accumulation, two-week-old plants were used to detect the levels of ROS accumulation under oxidative stress conditions. First, we determined the O_2_·¯ levels using nitroblue tetrazolium (NBT) staining, and the accumulation of H_2_O_2_ by 3,3-diaminobenzidine (DAB) staining. As shown in Fig. 5A and C, under normal conditions, no significant difference in color was observed between the three transgenic tobacco lines and WT. Oxidative stress increased the accumulation of both O_2_·¯ and H_2_O_2_, as lighter brown and blue precipitates were detected in the three transgenic lines. Quantification of H_2_O_2_ levels (Fig. 5B) and O_2_·¯ production rates (Fig. 5D) produced the same results as the staining.

**Fig 6:**
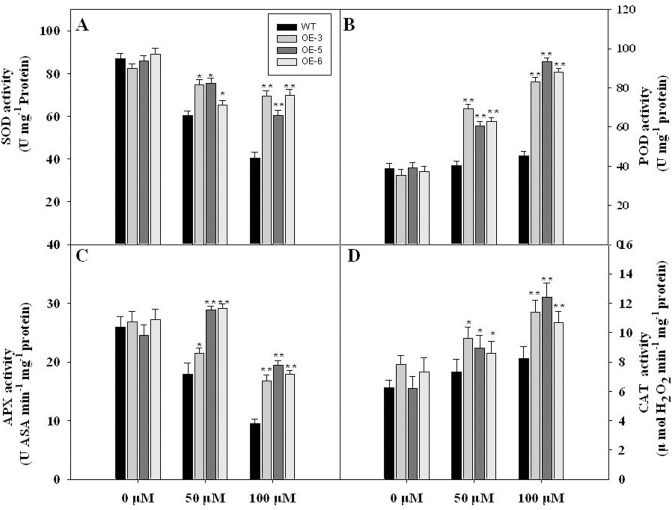
The total activities of the antioxidant enzymes in transgenic plants and WT under MV treatment. **(A)** Superoxidase dismutase, SOD; **(B)** Guaiacol peroxidase, POD; **(C)** Ascorbate peroxidase, APX; **(D)** Catalase, CAT. Values are the average of three replicates ± SE. “*” and “**” indicate significant differences between different plants subjected to the same treatment at the P < 0.05 and P < 0.01 levels, respectively.

We examined the activity of antioxidative enzymes, including ascorbate peroxidase (APX), catalase (CAT), superoxide dismutase (SOD), and peroxidase (POD) in *TaFBA1*-overexpressing transgenic lines and WT plants. Under normal conditions, no significant difference was found in the activities of the antioxidative enzymes examined. Oxidative stress changed their activities with different patterns. In general, the transgenic plants had higher antioxidative enzyme activity than WT under oxidative stress. For instance, the transgenic lines had significantly higher SOD activity, exhibited by 71.69, 49.51 and 73.0% increases compared with WT tobacco plants under 100 μM MV stress (Fig. 6A). A similar trend was found in the activity of APX, CAT and POD under MV stress.

### Expression profiling pathway antioxidant-related genes in transgenic plants

We examined the expression of some known genes encoding antioxidative enzymes in tobacco plants, including **AB093097** (*NtSOD*), **U93244** (*NtCAT1*), **AU15933** (*NtAPX1*), **D85912** (*NtAPX2*), **AB041518** (*NtGPX*), **AF454759** (*NtCA*) and **AJ309006** (*NtRbohD*). *NtSOD* encodes SOD in tobacco [28], whereas *NtCAT1*, *NtAPX1*, *NtAPX2* and *NtGPX* encode catalase, cytosolic ascorbate peroxidase, chloroplastic ascorbate peroxidase and glutathione peroxidase, respectively. All of these enzymes use H_2_O_2_ as an electron acceptor to catalyze a number of oxidative reactions [29]. *NtCA*, which encodes carbonic anhydrase, displays antioxidant activity and functions in the hypersensitive defense response [30]. *NtRbohD*, a tobacco respiratory burst oxidase homolog, encodes an enzyme similar to mammalian NADPH oxidase, producing active oxygen species in elicited tobacco cells [31].

**Fig 7:**
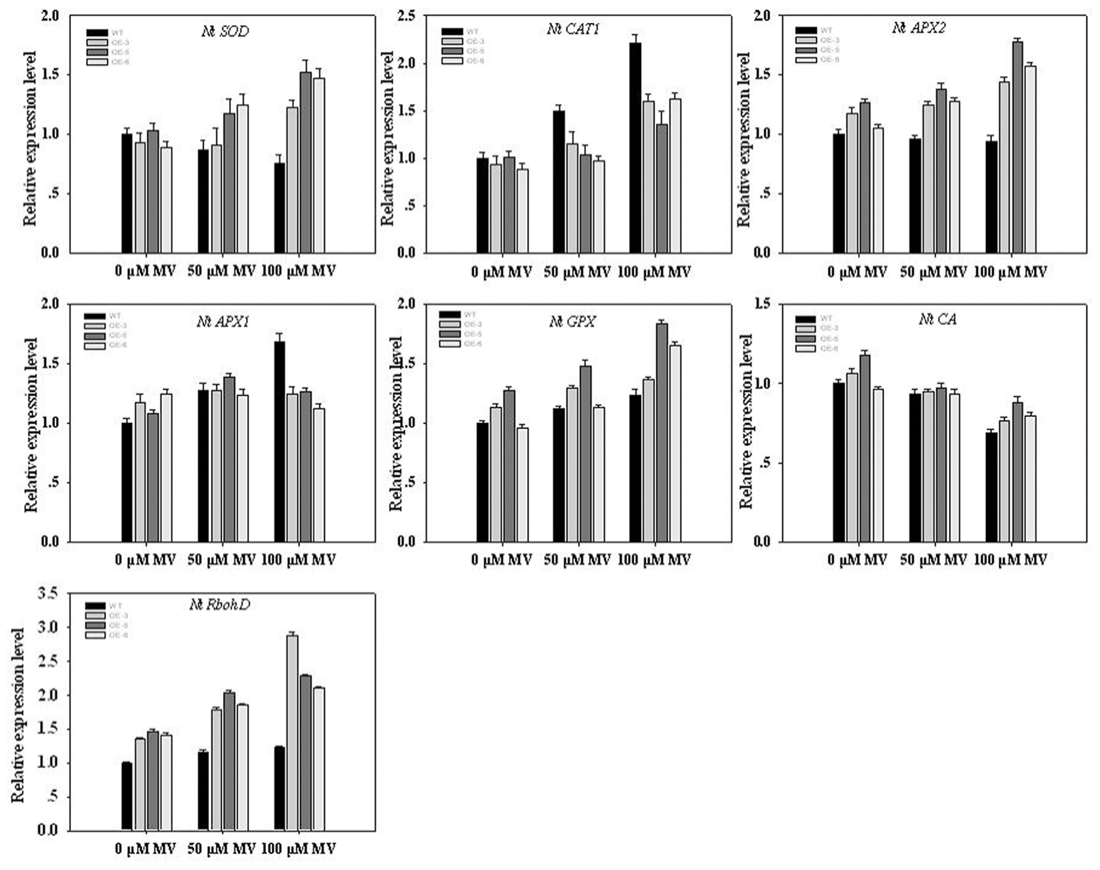
Expression of antioxidant-related genes in WT and transgenic plants under MV treatment by qPCR. Transcript levels of these genes in transgenic plants are indicated relative to the level of WT plants taken as 1, referring to the transcript of *actin* in the same samples. Each column represents the mean ± standard error of five replicates.

As shown in Fig. 7, oxidative stress upregulated the expression of some genes, e.g., *NtSOD*, *NtCAT1*, *NtAPX1*, *NtAPX2*, *NtGPX*, and *NtRbohD*, except *NtCA*. Compared with WT plants, the expression of the four upregulated genes (*NtSOD*, *NtAPX2*, *NtGPX*, *NtRbohD*) was higher in transgenic plants under oxidative stress conditions, with the most significant differences observed in the expression of *NtSOD* and *NtRbohD*. However, the expression of *NtAPX1* and *NtCAT1* was lower in transgenic plants under oxidative stress conditions compared with WT plants.

### The subcellular localization of TaFBA1 in plant cells

**Fig 8:**
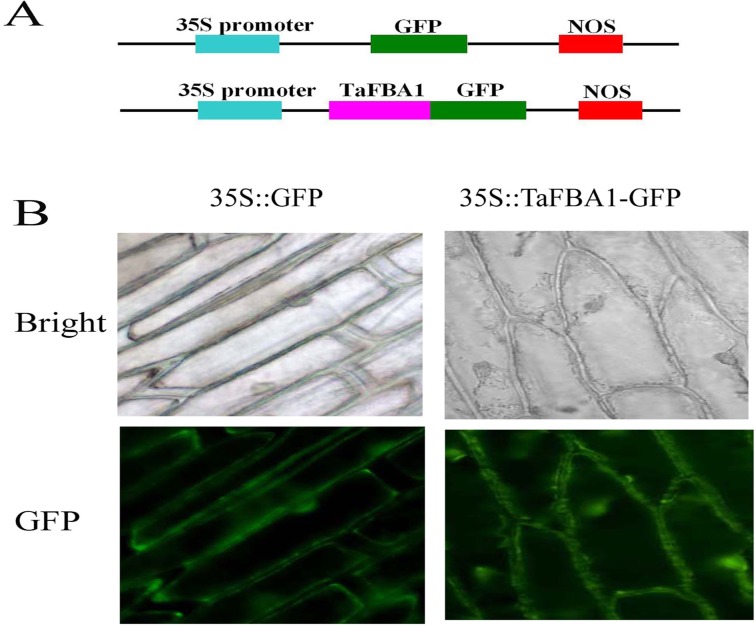
Subcellular localization of TaFBA1-GFP fusion protein in onion epidermal cells. (A) Schematic representation of the 35S::TaFBA1-GFP fusion construct and the 35S::GFP construct. The TaFBA1 coding region was fused upstream of the GFP coding region in the expression vector. (B) The transformed cells of the 35S::TaFBA1-GFP and 35S::GFP constructs were cultured in Murashige-Skoog (MS) medium at 28°C for 2 d and observed under a microscope.

To determine the subcellular localization of TaFBA1 in plant cells, a GFP reporter gene was fused to *TaFBA1* in a pBI121 vector. Onion epidermal cells were used to transiently express the fusion product 35S::TaFBA1-GFP using *Agrobacterium*-mediated transformation. As shown in Fig. 8A, the cells transformed with the 35S::GFP control construct without TaFBA1 showed GFP signals in whole cells. However, individual cells transformed with 35S::TaFBA1-GFP exhibited green fluorescence predominantly in the nucleus, cytoplasm and plasma membrane of onion epidermal cells (Fig. 8B), indicating that the TaFBA1 protein was localized in the nucleus, cytoplasm and plasma membrane.

## Discussion

### The F-box proteins are involved in plant responses to (a)biotic stresses

Some F-box proteins are involved in plant hormone signaling and responses to (a)biotic stresses [8, 32, 33]. The F-box protein DOR functions as a novel inhibitory factor for ABA-induced stomatal closure under drought stress in *Arabidopsis* [12]. The *Arabidopsis thaliana* F-box protein AtFBP7 is required for efficient translation during temperature stress [34]. Some studies have shown that the expressions of several F-box protein genes were regulated by abiotic stress. Jain *et al*. [35] found that the expression of 23 F-box proteins was upregulated or downregulated to salinity stress in rice. The F-box protein, *CarF-box1*, was also upregulated by salt and drought stress in chickpea [36]. The accumulation of mRNA from *PvFBS1*, a putative F-box gene from *Phaseolus vulgaris*, responds to wound and osmotic stress and the application of methyl jasmonate (MeJA), salicylic acid (SA) and ABA [13]. Yan et al. [33] found that the overexpression of the *MAIF1* gene reduces abiotic stress tolerance and promotes root growth in rice. However, TdRF1, a wheat RING ubiquitin ligase, has a protective role against cellular dehydration [37].

We previously cloned a new F-box protein gene, named *TaFBA1* (GenBank accession no. JN038382), from wheat, and we found that the overexpression of *TaFBA1* enhanced the tolerance of the transgenic tobacco plants [14]. Analysis of the *TaFBA1* promoter sequence demonstrates that *TaFBA*1 may function in multiple biological processes (Table 2). The results in Fig. 1 show that the expression of *TaFBA1* was upregulated by oxidative stress in wheat. Previous reports and those in this paper suggest that F-box proteins were involved in various stress responses of plants, but the underlying mechanisms are far from clear.

Oxidative stress is the most universal second stress involved in almost all stress conditions [5]. Here, the characteristics and involvements of *TaFBA1* in oxidative stress tolerance were studied using the transgenic tobacco plants that overexpressed *TaFBA1*.

### Overexpression of TaFBA1 enhances the oxidative tolerance in transgenic tobacco plants

The active component of MV, an herbicide called paraquat (1,10-dimethyl-4,40-bipyridinium dichloride), exerts its phototoxic effects on plants by transferring electrons from photosystem I to molecular oxygen, resulting in the accumulation of ROS in chloroplasts [38]. MV is a source of oxidative stress, and the damage caused by MV treatment under illumination is known as MV-mediated photooxidative damage [39]. In the current study, MV was also used in different developmental stages of the tobacco plants to induce oxidative stress.

In numerous crop plants, seed germination and early seedling growth are highly susceptible to abiotic stresses [40]. In this study, the seed germination of the transgenic lines was significantly higher than that in the WT under oxidative stress conditions (Fig. 2A, B). The transgenic tobacco seedlings were still alive and green, whereas WT tobacco leaves showed serious chlorosis under 10 μM MV treatments (Fig. 3A). The results in Figs. 2 and 3 indicated that the transgenic plants showed longer primary root and higher fresh weight than WT under MV stress. These results indicate that *TaFBA1* may be associated with the maintenance of seed germination and early seedling growth in plants under stress conditions.

The visible injury of leaf disks by MV treatment is often used to assay the tolerance of plants to oxidative stress [41]. In the present study, the in vitro experiment with leaf disks showed that only partial necrosis was observed at the boundaries of the leaf disks of the transgenic plants, whereas severe necrosis was observed in the leaf disks of WT plants (Fig. 3C). This result was further confirmed by measuring the chlorophyll content in the leaf disks after MV treatment (Fig. 3E). These results suggest that overexpression of TaFBA1 enhances the oxidative tolerance of transgenic tobacco.

Stress-induced changes are frequently related to an increase in membrane permeability, affecting membrane integrity and cell compartmentation under stress conditions. MDA, the product of lipid peroxidation caused by ROS, is generally used to evaluate ROS-mediated injuries in plants [42]. Electrolyte leakage through cell membranes is commonly considered to be an index of membrane damage or deterioration. We also quantified the MDA content and relative electrical conductivity as a measure of cellular damage in response to MV stress. Transgenic plants showed significantly lower MDA levels in response to MV treatments (Fig. 3F), together with the less relative electrical conductivity in transgenic plants (Fig. 3G). These results suggest that transgenic plants experienced less lipid peroxidation and had a greater capability to maintain their membranes’ stability under oxidative stress.

Derivatization of protein carbonyls with DNPH followed by immunoblotting with an anti-DNP antibody is a sensitive and specific method for the detection of oxidatively modified proteins and is generally regarded as an indicator of oxidative stress [17]. The lower amounts of accumulated protein carbonylation products observed in transgenic plants compared with the WT under both normal and oxidative stress conditions (Fig. 4) suggested less oxidative damage to proteins in transgenic plants.

### The enhanced oxidative stress tolerance of transgenic plants may be related to higher antioxidant enzyme activities via the upregulated expression of some antioxidant genes

Plants have evolved antioxidative systems to maintain ROS at low and steady-state levels, in which some antioxidative enzymes play essential roles in ROS scavenging and protect cells from oxidative damage [3]. Fig. 5 shows that the ROS accumulation of transgenic plants was lower than WT at MV treatment. Meanwhile, the activities of antioxidant enzymes, such as SOD, CAT, POD and APX, were higher in transgenic lines than in WT (Fig. 6). It is well known that SOD is the major O_2_·¯ scavenger and its enzymatic action results in H_2_O_2_ and O_2_·¯ formation. The produced H_2_O_2_ is then scavenged by CAT and POD [43]. Our results suggested that TaFBA1 is involved in ROS scavenging, especially under stress conditions. Furthermore, we detected the mRNA level of some genes of antioxidant enzymes. Overexpressing TaFBA1 in tobacco greatly increased the expression of some antioxidant genes compared with WT plants (Fig. 7). From these results, we speculated that the overexpression of TaFBA1 improves the antioxidant defense system by regulating the expression of the antioxidant genes, which in turn protect transgenic lines against oxidative stress.

SOD, CAT, APX and POD are effective intracellular enzymatic antioxidants that are localized in different cellular compartments, such as chloroplasts, mitochondria, peroxisomes and cytosol [3]. Our results as shown in Fig. 8 indicate that TaFBA1 was localized in the nucleus, cytoplasm and plasma membrane, suggesting the possibility that TaFBA1 may be involved in regulating the activity and/or gene expression of antioxidant enzymes in different organelles, but the mechanism of regulation should be explored in future works.

In conclusion, our results suggest that the overexpression of *TaFBA1* confers enhanced tolerance to oxidative stress with the tobacco plants. The enhanced tolerance of oxidative stress may be associated with higher activities of the antioxidant enzymes, which may have resulted from the regulation of the antioxidant gene expression. This is important for understanding the functions of F-box proteins in plant tolerance to multiple stress conditions.

## Supporting Information

S1 File The data of results in the article. (XLS)
